# Supplementary material for: Caries Experience and Risk Indicators in a Portuguese Population: A Cross-Sectional Study
Source: Int J Environ Res Public Health. 2023 Jan 31;20(3):2511. doi: 10.3390/ijerph20032511 (PMC9915840; doi:10.3390/ijerph20032511)
Supplement: Supplementary file 1 [file ijerph-20-02511-s001.zip › ijerph-2190470-supplementary.pdf]

Supplementary Materials: Table S1

Table S1. Univariate logistic regression analysis of sociodemographic, behavioral, anthropometric and oral hygiene variables towards the outcome variable 'caries presence' (N=9,349)

**Variable**		**OR (95% CI)**	**P**
Gender	Female	1	-
	Male	1.04 (0.90-1.20)	0.617
Age (years)	18-24	1	-
	25-44	3.27 (2.72-3.92)	**< 0.001**
	45-64	4.30 (3.55-5.21)	**< 0.001**
	≥65	4.16 (3.24-5.33)	**< 0.001**
Education level	No education	1	-
	Elementary	0.55 (0.07-4.07)	0.562
	Middle	0.27 (0.04-1.99)	0.199
	Higher	0.19 (0.03-1.41)	0.105
Occupation	Student	1	-
	Employed	4.20 (3.57-4.95)	**< 0.001**
	Unemployed	4.99 (3.75-6.65)	**< 0.001**
	Retired	5.11 (4.00-6.52)	**< 0.001**
Smoking habits	Non-smoker	1	-
	Smoker	1.06 (0.90-1.25)	0.495
Alcohol consumer	No	1	-
	Yes	0.87 (0.76-1.01)	0.068
BMI	Underweight	1	-
	Normal weight	1.01 (0.81-1.25)	0.940
	Overweight	2.14 (1.68-2.73)	**< 0.001**
	Obese	2.08 (1.58-2.74)	**< 0.001**
Last dental visit	< 1 year	1	-
	1-2 years	1.24 (0.99-1.54)	0.055
	3-4 years	1.07 (0.87-1.31)	0.515
	≥ 5 years	1.42 (1.14-1.77)	**0.002**
	Never	0.49 (0.30-0.79)	**0.004**
Consultation motive	Routine	1	-
	Aesthetics	1.28 (0.89-1.84)	0.180
	Pain	1.43 (1.16-1.77)	**0.001**
	Functional	1.48 (1.25-1.74)	**< 0.001**
	Other	0.95 (0.62-1.44)	0.797
Toothbrush frequency	2-3 times/daily	1	-
	1 time/daily	1.28 (1.05-1.58)	**0.017**
	2-6 times/weekly	0.89 (0.53-1.51)	0.671
	Never	3.65 (1.35-9.88)	**0.011**
Dental floss usage	Yes	1	-
	No	1.02 (0.88-1.19)	0.760
Mouthwash usage	Yes	1	-
	No	0.89 (0.77-1.02)	0.100
Teeth health perception	Excellent	1	-
	Very good	1.51 (0.99-2.29)	0.056
	Good	2.39 (1.62-3.52)	**< 0.001**
	Weak	4.14 (2.77-6.19)	**< 0.001**

	Very bad	3.93 (2.56-6.03)	**< 0.001**
Gums health perception	Excellent	1	-
	Very good	1.13 (0.79-1.63)	0.498
	Good	1.67 (1.21-2.32)	**0.002**
	Weak	2.01 (1.42-2.85)	**< 0.001**
	Very bad	2.21 (1.43-3.42)	**< 0.001**
Comorbidity	No	1	-
	Yes	0.94 (0.81-1.08)	0.403

Abbreviations: BMI – Body Mass Index; CI – Confidence Interval; OR – Odds Ratio.
